# Applications of Various Types of Nanomaterials for the Treatment of Neurological Disorders

**DOI:** 10.3390/nano12132140

**Published:** 2022-06-22

**Authors:** Abdul Waris, Asmat Ali, Atta Ullah Khan, Muhammad Asim, Doaa Zamel, Kinza Fatima, Abdur Raziq, Muhammad Ajmal Khan, Nazia Akbar, Abdul Baset, Mohammed A. S. Abourehab

**Affiliations:** 1 Department of Biomedical Sciences, City University of Hong Kong, 83 Tat Chee Avenue, Kowloon, Hong Kong SAR, China; awaris@bs.qau.edu.pk (A.W.); muhamasim5-c@my.cityu.edu.hk (M.A.); 2 Department of Biotechnology and Genetic Engineering, Hazara University Mansehra, Mansehra 21300, Pakistan; asmat6597@gmail.com (A.A.); naziasaadiq@gmail.com (N.A.); 3 CAS Key Laboratory of Standardization and Measurement for Nanotechnology, CAS Center for Excellence in Nanoscience, National Center for Nanoscience and Technology, No. 11 Zhongguancun Beiyitiao, Beijing 100190, China; atta2021@nanoctr.cn (A.U.K.); kinzafatima251@gmail.com (K.F.); 4 Department of Environmental Engineering, Institute of Urban Environment, CAS, Xiamen 361021, China; doaazamel@gmail.com; 5 Department of Physical Chemistry, University of Chinese Academy of Sciences, Beijing 100049, China; 6 Department of Physics, Bacha Khan University Charsadda, Charsadda 24420, Pakistan; abdurraziqbkuc@gmail.com; 7 Divison of Life Sciences, Center for Cancer Research and State Key Laboratory of Molecular Neurosciences, The Hong Kong University of Science and Technology, Clear Water Bay, Hong Kong SAR, China; makhan@connect.ust.hk; 8 Department of Zoology, Bacha Khan University Charsadda, Charsadda 24420, Pakistan; drabdulbaset@bkuc.edu.pk; 9 Department of Pharmaceutics, Faculty of Pharmacy, Umm Al-Qura University, Makkah 21955, Saudi Arabia; 10 Department of Pharmaceutics and Industrial Pharmacy, Faculty of Pharmacy, Minia University, Minia 61519, Egypt

**Keywords:** nanotechnology, nanomaterials, neurological disorders, blood-brain barrier

## Abstract

Neurological disorders (NDs) are recognized as one of the major health concerns globally. According to the World Health Organization (WHO), neurological disorders are one of the main causes of mortality worldwide. Neurological disorders include Alzheimer’s disease, Parkinson′s disease, Huntington′s disease, Amyotrophic lateral sclerosis, Frontotemporal dementia, Prion disease, Brain tumor, Spinal cord injury, and Stroke. These diseases are considered incurable diseases because no specific therapies are available to cross the blood-brain barrier (BBB) and reach the brain in a significant amount for the pharmacological effect in the brain. There is a need for the development of strategies that can improve the efficacy of drugs and circumvent BBB. One of the promising approaches is the use of different types of nano-scale materials. These nano-based drugs have the ability to increase the therapeutic effect, reduce toxicity, exhibit good stability, targeted delivery, and drug loading capacity. Different types and shapes of nanomaterials have been widely used for the treatment of neurological disorders, including quantum dots, dendrimers, metallic nanoparticles, polymeric nanoparticles, carbon nanotubes, liposomes, and micelles. These nanoparticles have unique characteristics, including sensitivity, selectivity, and the ability to cross the BBB when used in nano-sized particles, and are widely used for imaging studies and treatment of NDs. In this review, we briefly summarized the recent literature on the use of various nanomaterials and their mechanism of action for the treatment of various types of neurological disorders.

## 1. Introduction

Neurodegenerative disorders (ND) are the most devastating and challenging disorders of the central nervous system (CNS) and are recognized as a major threat to public health [1]. ND refers to the loss of structure or functions of the neuron. There are different types of ND, including stroke, Alzheimer′s, Parkinson′s, Huntington′s, and prion diseases. The pathophysiology of each disease is different. Some NDs lead to memory and cognitive impairment, while some affect the ability of the person to speak, move, and breathe [2]. According to the World Health Organization report, in 2019, in the list of top 10 leading causes of diseases globally, stroke is the second leading cause of death, followed by Alzheimer′s disease and other dementias [3]. In developing countries, neurological disease cases increase each year with the progressive rise in life expectancy. The cause of most neurological diseases is well known, and many studies have been done on its treatment. The central nervous system is a vulnerable and complex system that creates complications in diagnosing and treating neurological diseases [4,5]. Some barriers create complications for therapeutic intervention, and the functions of these barriers are to regulate the molecular exchange between blood and the brain. The barriers are made up of glial cells and endothelial cells in the brain. The blood-brain barrier (BBB) is the main gateway of the system as it administers the drugs′ access to the brain [6].

NDs are considered incurable diseases, and the prevalence of these diseases is rapidly rising. The majorities of these diseases have no specific effective therapies because of the inability of drugs to cross the BBB to reach the brain and being available in an amount that is high enough for a pharmacological effect in the brain. The BBB is one of the challenges—but not the only challenge—for treating neurological disorders. Approximately 95% of drugs cannot cross the BBB because of their molecular property. The central nervous system is restricted by BBB and blood-cerebrospinal fluid. Due to the restrictive nature of the BBB, lipophilic drugs with molecular weight of <500 Da can cross, while traditional drugs cannot meet this requirement [7,8]. The BBB is highly semipermeable, which leads to limiting the entry of therapeutic drugs to enter the CNS, and this property of the BBB is considered one of the main challenges in the development of modern medicine. Photothermal and photodynamic therapies are considered alternative approaches and are used to treat various NDs due to their ability to bypass the BBB. However, these therapies have side effects, including damage to tissues (especially skin tissues) and photosensitization [9,10]. Therefore, there is an urgent need for the development of potential therapeutic agents that can cross the BBB with no side effects to combat these diseases. However, a deep understanding of the mechanism and causes are necessary for the effective treatment of each disease [2,11].

There is a need for the development of therapeutic strategies that can overcome the BBB and also can improve its efficacy. Researchers are continuously working on the development of delivery strategies of therapeutics to solve this problem [12,13]. Different strategies that can overcome this problem have been developed. Among these approaches, the nano-based approach is at the core of these advances in the delivery of therapeutics [14]. Nanotechnology uses different types of engineered nanomaterial and nanoparticles with a size of 1–100 nm in at least one dimension [12,15]. Nanotechnology and nanomaterials open new avenues in biomedical science, as multiple nanoparticles have been applied in brain studies and research, including quantum dots (QDs), polymeric nanoparticles, micelles, metallic nanoparticles, etc. [16]. These nano-scale materials possess unique characteristics such as small size due to which they can interact with the biological system at the molecular level, a high surface to volume ratio that can be mono or multifarious with surface modification, and good stability. On the other hand, the application of chemotherapy and drugs may lead to side effects such as anemia, alopecia, gastric irritation, neurotoxicity, and loss of appetite. Encapsulation of drugs in metallic nanoparticles, for example, silver, gold, and metal oxides (magnetic), aids in overcoming the complications of medications [17]. In the past decade, different types and shapes of nanomaterials have been widely used to treat different types of NDs [18,19]. Researchers have achieved remarkable progress in the field of nanotechnology in nanomedicine and biomedical sciences, especially in neurosciences. In this contribution, we briefly discussed the types of nanomaterials and the advancement of nanotechnology in the field of neurosciences.

## 2. Types of Nanomaterials Used for the Treatment of Neurological Disorders

### 2.1. Quantum Dots

QDs are unique nanoparticles that have a wide range of applications as a treating agent for various neurodegenerative diseases. QDs have unique characteristics such as sensitivity and selectivity when used in nano-sized particles. Several research articles have been published studying the QDs applications in diagnosing and treating Alzheimer′s disease (AD). It has been proved that QDs have the ability to cross the BBB, whereas potential toxicity could be further evaluated [16].

Furthermore, QDs have unique optical properties, enriching their widespread use in biomedical applications. However, great concern towards QDs has been taken in the last years due to their potential toxicity. Herein, neurotoxicity increases in the case of the distribution of QDs mass production in the nervous system. Due to the very small size of QDs particles, they can cross the BBB or move with the blood circulation entering the brain. Nevertheless, the interactions between QDs and the nervous system cells and tissues are not clear yet; the obvious is the neurotoxicity, which includes oxidative stress, increased Ca^2+^ levels in the cytoplasm, autophagy—which damages in vitro neural cells, impairment of synaptic transmission, and loss of plasticity, as demonstrated in tested animals [20]. QDs are basically nanocrystals in nano-sized structures that can emit light, which gives them unique optical properties. QDs have fluorescent light of several wavelengths, which gives them brightness and resistance to bleaching. Therefore, QDs can visualize brain structures and mechanisms of their functions as well as be applied for drug delivery purposes [21]. In the past few years, in vivo imaging of biological functions has been performed by near-infrared (NIR) fluorescence imaging techniques. NIR (700–900 nm) could be demonstrated for sensitive and accurate detection techniques. Then there are the QDs semiconductors, which could be utilized in detection due to their electronic, magnetic, optical, and structural characteristics, which are different from bulk materials. QDs could be a contrast agent for optical imaging, especially in deep tissue imaging [22]. Moreover, though emerging evidence indicates that the pathogenesis of Parkinson′s disease is strongly correlated to the accumulation and transmission of α-synuclein (α-syn) aggregates in the midbrain, no anti-aggregation agents have been successful at treating the disease in the clinic. Graphene quantum dots (GQDs) inhibit the fibrillization of α-syn and interact directly with mature fibrils, triggering their dis-aggregation. Moreover, GQDs can rescue neuronal death and synaptic loss, reduce Lewy body and Lewy neurite formation, ameliorate mitochondrial dysfunctions, and prevent neuron-to-neuron transmission of α-syn pathology provoked by α-syn preformed fibrils. We observe, in vivo, that GQDs penetrate the BBB and protect against dopamine neuron loss induced by α-syn preformed fibrils, Lewy body/Lewy neurite pathology, and behavioral deficits [23]. Further studies worked on GQDs as labeling agents for stem cells, which develop little cytotoxicity. They were studied on human neural stem cells to determine their uptake and biocompatibility. The results came up with no significant change in the viability, metabolic activity, proliferation, and differentiation potential of human neural stem cells after the treatment with GQDs. Furthermore, GQDs have been taken up into the neural stem cells via endocytosis [24]. GQDs could be considered promising treatment agents for NPC and other related diseases [21]. Compared to large graphene oxide nanosheets, graphene oxide quantum dots (GOQDs), as nanozymes, could be proved to efficiently decrease the reactive oxygen species (ROS) and H_2_O_2_ in 1-methyl-4-phenyl-pyridinium ion (MPP+)-induced PC12 cells. Furthermore, GOQDs have the ability to reduce apoptosis and α-synuclein and mitochondrial damage in zebrafish treated with MPP+. To sum up, biocompatible GOQDs have a high potential for human health by diminishing oxidative stress signals and reducing neurotoxicity [25].

Silicon nanocrystals provide excellent imaging capabilities for toxic heavy-metal-based QDs. However, understanding the toxicity developed by silicon quantum dots (SiQDs) is mandatory for estimating its potential. There are only a limited number of studies on the biocompatibility of SiQDs and no obvious systematic progression from clinical studies on small-animal to large-animal. The nano-construct of SiQDs and FDA-approved materials are applied intravenously in mice and monkeys, and the results demonstrated no toxicity in both mice and monkeys in their behavior, blood, and body mass at a dose of 200 mg/kg. The drug formula did not biodegrade as well, as high levels of silicon were accumulated in the liver and spleen of mice after three months of the treatment and nothing showed in monkeys [23].

Another study targeted carbon dots (CDs) and showed their promising effects for various biomedical applications such as bio-imaging, treatment of brain tumors, and neurodegenerative diseases. CDs showed unique properties like biocompatibility and small sized-particles less than 10 nm, enabling them to enter the BBB. They have different optical properties and stability toward the light, which lets them acquire ideal characteristics for applications in several scientific fields [26]. The BBB is the physiological checkpoint that restricts the passage of molecules present in the blood into the central nervous system. One of the scientists′ serious challenges is delivering drugs and active materials across the BBB. Therefore, developing novel materials and methodologies to address this challenge is vital for diagnosing and treating brain diseases. In a study, bio-conjugated and functionalized QDs were revealed to be outstanding nano-vectors and fluorescent probes for the transmission across the BBB for treating brain tumors and diseases [27]. Previous studies on photoluminescent 9-nm diameter QDs with a CdSe core, a ZnS shell, and a negatively charged compact molecular ligand coating (CL4) selectively target neurons instead of glia. On the other side, current research studies focus on the explanation for the selective delivery in neurons. Three zwitterionic QD coatings differ in the regions of positive or negative charges, as well as a positively charged (NH2) polyethylene glycol (PEG) coat, which enriches them with the ability to deliver the cell-membrane-penetrating chaperone lipopeptide JB577 (WG(Palmitoyl) VKIKKP9G2H6) to the individual cells in neonatal rat hippocampal slices. The results confirmed the preferential uptake in neurons and vice-versa in glia, which showed no uptake, and can be explained due to the negatively charged regions on the coating of QDs. Moreover, the treatment was not effective in the astrocytes and microglia cells, and researchers suggested the administration of a histidine-tagged green fluorescent protein (eGFP-His6) to hippocampal slices to enhance the neuronal uptake [28]. In another in vivo study using graphene-Quantum dots (GQDs) in NPC, GQDs were revealed, which can possess little long-term toxicity and which can be neglected as they have the penetration capability of the BBB. The treatment using GQDs decreases the cholesterol aggregation in the lysosome via expressed interactions [29].

**Figure 1:**
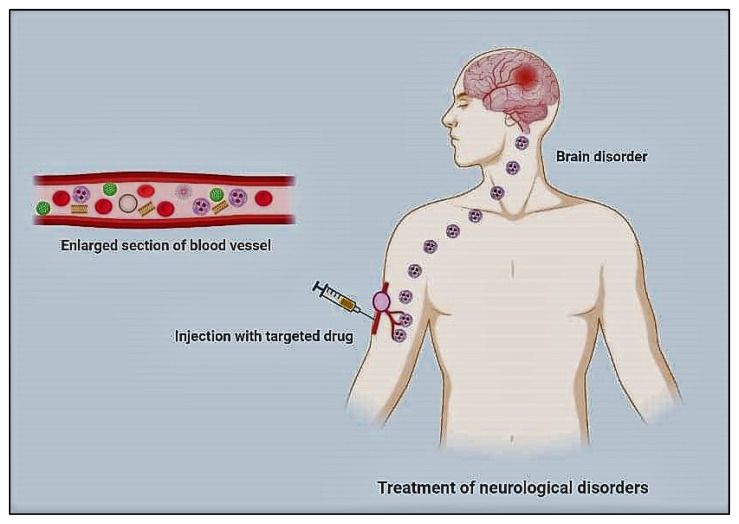
Schematic illustration of drug delivery system for the treatment of neurological disorders.

Further studies performed on the selenium-doped carbon quantum dots (Se-CQDs) showed their ability to diminish reactive oxygen species and that they have been applied to efficiently ameliorate secondary injury in TSCI. The results demonstrated the good biocompatibility and remarkable protective effect of Se-CQDs against H_2_O_2_-induced oxidative damage in astrocytes and PC12 cells [30]. The schematic process of the drug delivery system is shown in Figure 1 for the treatment of neurological disorders.

### 2.2. Metallic Nanoparticles

Metallic nanoparticles can be fabricated, modifying their shape and size, and can link to various types of chemical functional groups [31]. These modifications and linkings allow them to attach and bind with various ligands, including drugs, antibodies, peptides, and polymers. Different types of metallic nanoparticles are used as a carrier for brain-targeted therapy. These nanoparticles include FeONPs, AgNPs, Gadolinium metallofullerene NPs, Ultrasmall gadolinium oxide NPs, Ce_2_O_3_ NPs, ZnONPs, AuNPs, and PtNPs. Metallic nanoparticles are also used for the imaging of CNS [9,32].

Metallic nanoparticles have shown their outstanding role in biomedical applications [13,33,34,35]. Currently, these materials can be manufactured and functionalized with groups that facilitate their conjugation with antibodies, drugs, and ligands—these modifications and functionalization open new avenues of potential applications in magnetic separation, biotechnology, and drug delivery. Furthermore, various imaging models have been promoted over the years, such as CT, MRI, PET, ultrasound, optical imaging, and SERS, which are good tools for several disease images [36]. Nanomaterials have been used widely in recent years for the diagnosis, imaging, and treatment of various disorders. However, nanoparticles possess potential hazards and neurotoxicity to the CNS, such as autophagy, oxidative stress, and lysosomal dysfunction via possible mechanisms [37].

Moreover, nano-delivery methods can effectively cross the BBB and reach remote regions in the brain. However, a deep understanding of developing the long-term toxicities is the primary question of scientists, which needs to be addressed [38]. Research articles on biogenic metal or metal oxide nanoparticles are limited, especially those for treating Alzheimer’s disorder. In addition, their functionalization is suggested to develop their therapeutic potential for treating neurodegenerative diseases [39]. Furthermore, metal nanoparticles are excellent carriers and therapeutic materials that aid in biomedical applications due to their unique characteristics. They have physicochemical properties, which enrich their possibility to be used in many fields in science.

Moreover, metal nanoparticles can be manufactured while modulating their size and morphology as well as functionalization with other ligands, which widen their applications and increase their efficiencies [32,40]. Additionally, biodegradable biomaterials that have been functionalized offer promising ways to address that question for solutions due to their unique properties, such as the ability to respond to external stimulation. These unique properties enriched their applications in neuro-sensing, neuro-imaging, specific targeting, drug delivery, and treatment of hyperthermia [41]. Though nanoparticles possess good physical and chemical properties, which demonstrate a wide range of applications for CNS, they may develop neurotoxicity owing to cell necrosis, immune response, and free radical formation [42].

### 2.3. Dendrimers

Optical dendrimers provide the combined advantages of particle-defined structure and the functionalized surface, which makes them outstanding in the application of neuroscience. It is important to mention that the most recent discovery based on dendrimers is the agent of MRI contrast, which improves the bloodstream′s visualization [43]. As has been well defined, dendrimers are versatile polymeric compartmentalization with unique sizes and physicochemical properties and resemble biomolecules. Therefore, dendrimers can be used as antibacterial, antiviral, and anticancer agents. In addition, they are playing an outstanding role in drug delivery and targeting by special interaction between host–guest binding motifs. Dendrimers may be used as glycol carriers for targeting tissues in the diagnosis and treatment of malignant disorders. They also present antigens for vaccines, such as peptides [44]. Dendrimers are nanostructured materials that have branched layers that look like onion skin in their morphology. These nano-sized structures grow in layers producing branches similar to as in globular proteins. The outer layers contain a high number of functional groups that provide these nanostructures with unique features for applications in the medical fields of science as nanocarriers for drugs, nucleic acids, and proteins. Furthermore, they can be used in imaging applications such as a novel dendrimer-based theranostic nanodevice for imaging and treatment of carcinoma [45]. Recently, dendrimers are invading not only chemistry but also biology, medicine, and physics, and their fascinating results are growing day by day [46]. The bioagents or biomolecules may be physically/chemically adsorbed on the surface of dendrimers or encapsulated into the core of dendrimers. Herein, the elevated number of functional groups on the dendrimer surfaces aid in the attachment of the targeting groups, and further, they interfere in the functionality and toxicity of dendrimers [47]. Most drugs cannot achieve the desired bioavailability when used for brain disorders due to the selective permeability in the brain and low water solubility. Therefore, scientists innovated the drug delivery systems for targeting drugs and directing their entry into the target organs in the body. The nano-polymeric structures of dendrimers with ordered particle size and morphology make them ideal agents for biomedical applications. There are several examples of dendrimers such as Glyco, PEGylated, poly(propylene imine), poly(amidoamine), polyether-copolyester, and pH dendrimers. These kinds of dendrimers can carry the drug molecules by physical or chemical interactions, whereas pH dendrimers alter the ionic exchange in the tumor area in the brain [48].

Bio conjugation of poly(amido)amine dendrimer and streptavidin adapter was recently studied in vitro on murine or porcine models. In vivo studies on mice demonstrated good permeation of dendronized streptavidin into the central nervous system [30]. In a further study, the dendrimers viability has been studied in human astrocytes, and their uptake was explained. The results revealed the detection of dendriplexes inside the brain using a sensitive imaging system of fluorescent imaging in vivo (IVIS Lumina) and by confocal microscopy analysis of sections of OCT-embedded tissues. Moreover, the effective permeability of 2G-(SNMe3I)11-FITC dendrimer siRNA into the brain passes through the BBB [49]. Another in vitro and in vivo study tested the effect of targeting agent conjugation if they would increase or change the uptake by the brain and organs—or not. Herein, mannose was conjugated to the surface of multifunctional D4-OH in the presence of atom-economical and orthogonal Cu (I)-catalyzed alkyne–azide cycloaddition. It was revealed that the conjugation of mannose modifies the mechanism of dendrimers internalization, as stated in the in vitro results.

Furthermore, the brain uptake was also studied on a rabbit model via confocal microscopy and fluorescence spectroscopy [50]. A study targeted the development of the oral drug N-Acetyl-l-cysteine (NAC), which is widely used in acetaminophen poisoning and further used for the treatment of some neurological diseases like cerebral palsy. The results showed its stability for 6 h in the gastrointestinal tract, good permeability in the epithelial layer during in vitro study, and in vivo absorption in rats while conjugation with Capmul (glycerol monocaprylate) as an enhancer for the penetration [51]. Another study focuses on the preparation and characterization of hydroxyl poly(amidoamine) generation-6 dendrimers, which have permeability into the brain as well as long stability in the blood circulation. The conjugation of the drug minocycline has been performed on the mentioned dendrimers by enzyme linkages. The produced complex of the drug carried on dendrimers has been further investigated for antioxidant and anti-inflammatory activities in murine microglial cells. The in vivo results revealed a permeation of the prepared drug complex crossing the BBB [52]. A recent in vivo study used the rabbit model for applications of dendrimers for neuroinflammation and drug delivery without ligands. Here, neutral dendrimers moved towards the parenchyma and were highly localized in the glial cells in the brain injury areas. It was found that the uptake of dendrimers is dependent on the break-down severity of the BBB [53]. Dendrimers have invaded neuroscience applications as they have unique structural properties and morphologies, for instance; they are globular, highly branched, well-defined, have low polydispersity, nanosize-scale structure, and the existence of variable terminal functional groups that have the ability to conjugate with several ligands to be suitable for applications in various biological fields [54]. Additionally, dendrimers can be considered promising nanocarriers for central nervous system drugs owing to their unique features and wide applications [55].

### 2.4. Carbon Nanotubes

In nanomaterials, carbon nanotubes are a kind of nanomaterial that are gaining interest due to their electronic, intrinsic mechanical, and physico-chemical properties [56,57]. Carbon nanotubes (CNTs) can diagnose and treat neurological pathologies like Alzheimer’s and Parkinson’s disease. Approximately 24 million people around the globe suffer from Alzheimer’s and Parkinson’s diseases [58]. CNTs are classified into two nanotubes: first is a single-walled carbon nanotube (SWCNTs), and the other is a multi-walled carbon nanotube (MWCNTs) [59]. Most of the time, we use carbon nanotubes for drug delivery and bioimaging; due to this, we neglect its application as a therapeutic drug [60]. Because of the unique property and novel structure of carbon nanotubes, they have emerged as a promising option for tracking central nervous system diseases and their treatment. Advances in CNTs have made a significant contribution to therapeutic applications in different neuropathological disorders in vitro and in vivo [1]. CNTs are synthesized using techniques such as chemical vapor deposition (CVD), laser ablation, and arc discharge. Chemical vapor deposition is the most suitable and preferred method because it shows evidence of properties like electrical conductive capacity, strong mechanical property, morphological similarities to neurites, low cost, deposition, and scalability [61]. CNTs in their natural state do not dissolve in an aqueous solution; due to this property, their application in nanomedicine is complex. However, CNTs show their toxicity in their biological environment in clinical trials. However, their efficacy and side effects depend upon their exposure, dose, and route. Coating carbon nanotubes can minimize their toxicity to cells with surfactants, reducing their connection between cells and CNTs [62]. Metal-catalyzed prepared carbon nanotubes and the application of carbon nanotubes show free radical production, and peroxidative production, DNA damage, and inflammations are obstacles in the pathway of application of carbon nanotubes in the diagnosis and treatment of neurological disorders [63].

### 2.5. Polymeric Nanoparticles

Nanocarriers are fabricated from polymers, lipids, and carbon nanotubes, but polymer materials have good properties as they are stable, allow for many agents, and for controlling the drugs′ kinetic energy [64]. Polymers are safe to use in humans. Different kinds of polymers are used to synthesize nanoparticles for drug delivery to the central nervous system, such as are polysaccharides, proteins, amino acids, and polyesters [65]. Polymeric nanoparticles are made up of natural or synthetic polymers where the drug is loaded with a solid-state or in a solution. Polymeric nanoparticles are a promising method for drug delivery to the central nervous system. They protect the drug against enzymatic degradation, and help the active molecule reach its target site [66]. The most commonly used polymers are polyalkyilcyanoacrylate (PACA), which is used in nanoparticles for drug delivery to the central nervous system. PACA has been used for the treatment of tumors. A PACA nanoparticle is coated with the antitumor drug doxorubicin, which shows excellent tolerance against resistant tumors [67]. Different strategies have been made to cross the BBB to target nanoparticles to the central nervous system. Magnetic nanoparticles, nano gel, emulsifying wax, and ligand-based approaches are used [68]. Polymeric nanoparticles are the best alternative way of drug delivery due to their inherent biodegradability and nontoxicity [69]. The advantages of polymeric nanoparticles are their increased stability, delivery of a higher concentration, and because they can be easily incorporated. Lipid-based nanocarriers are recommended due to their natural-based condition, and because they are similar to the BBB membrane. Nevertheless, polymeric nanoparticles show a high capacity, and their surface is easily modified by targeting molecules; due to this, the polymeric nanostructured based system is the best alternative to drug delivery to the central nervous system [70].

### 2.6. Liposomes

Liposomes consists of lipid bilayers and are in the shape of spherical vesicles. The advantage of liposomes is that they are highly biocompatible, have low toxicity, and are without any side effects in the drug delivery system. Liposomes are made up of phosphatidylcholine and cholesterol [71,72]. For targeting the brain system, GABA, which contains liposomes, must consider two challenges: the BBB and microglia reaction. Osmotic shock temporarily opens the BBB to enhance the drug delivery through liposomes. GABA-based liposomes can target neurodegenerative diseases like anxiety, stress, epilepsy, and other physiological disorders like hypertension and heart failure [4,73].

Multiple methods have been proposed for the delivery of drugs to the brain. There are two families of transporters; the first is reversible, and the second one is irreversible nanoparticles. Liposomes and micelles are an example of reversible nanoparticles [74]. Theranostics is the new discipline in medicine that is simultaneously performed as a therapeutic and diagnostic function. Theranostic agents are molecules such as liposomes and micelles that can be used as a drug delivery vehicle in a protected and controlled manner. Using magnetic targeting of cells and tissues makes it more efficient by focusing on the activity of the pathological tissues, reducing unnecessary delivery and an excessive amount of the drug [4,75]. The theranostic approach is being used to target the brain because of the multitasking nature involved in constructing complex nanostructures like drug delivery vehicles to cross the BBB. A theranostic molecule like heat shock protein [HSP]-72 targeted liposomes has been shown to increase the efficiency of the treatment of neurological disorders [76].

### 2.7. Micelles

Displaying a variety of the arrangement along a chain, angle copolymers carry fresh blood to the old story of polymeric micelles. The angle chain structure brings about a few extraordinary highlights in micelles designs and prompts exceptional primary advances, possibly prompting new properties and applications. Hence, slope copolymer micelle structures and their advances form the perspective of delicate matter physical science [77,78]. Polymeric micelles are nanoscopic center/shell structures framed by amphiphilic block copolymers. The inborn and modifiable properties of polymeric micelles make them especially appropriate for drug conveyance purposes. The benefits and applications examined incorporate solubilization of ineffectively solvent atoms, supported delivery and size benefits, and insurance of embodied substances from debasement and digestion. The three most generally concentrated block copolymer classes are described by their hydrophobic squares and are poly(propylene oxide), poly(L-amino acid)s, and poly(ester)s. These three classes of square copolymers are investigated with numerous ebb and flow research instances in which definition procedures with polymeric micelles have been applied to the absolute most testing atoms in the drug business. The polymeric micelles utilized for drug conveyance in these models have shown a capacity to lessen poison levels, upgrade the conveyance to wanted natural destinations, and work on the remedial viability of dynamic drug delivery [79].

For the treatment of neurological diseases, many chemically synthesized drugs are used: cholinesterase inhibitors, Anti-Aβ regimens, and β-site peptide cleaving enzyme-1 (BACE1) inhibitors. These drugs are not considered as efficient for treatment due to their side effects [80]. As the brain is protected by barriers such as the BBB and blood-cerebrospinal fluid barrier (BCSFB), the drug molecules cannot move to the target site of the brain due to these barriers [81]. Nanocarriers are used for the treatment of central nervous system disorders. They are fabricated with features that control the drug delivery to the brain and increase the efficacy [82]. Polymeric micelles, with their unique properties, particle size, and shell structure, work as a drug delivery vehicle to the brain. The nature of the micelles is amphiphilic, as it can easily assemble itself in an aqueous medium; due to this, it protects the drug from the non-target cells [83].

Natural proteins derived from milk are used in the making of nanocarriers, such as lactoferrin (LF), which is expressed over the brain′s surface, through which the LF is attached to the receptor; due to this, it can easily pass through the BBB [83]. It was prepared by the solvent evaporation technique, and fabricated through covalent conjugation between lactoferrin. Micelles were utilized to encapsulate conjugated linoleic acid [84]. In vivo rhodamine micelles show better biodistribution in brain tissue, enhancing the active target capacity. It is concluded that lactoferrin and conjugated linoleic acid micelles are non-toxic, and they naturally activate the target nano platform, which provides a solution to Alzheimer′s disease [85].

## 3. Nanomaterials for the Treatment of Neurodegenerative Diseases

### 3.1. Alzheimer’s Disease (AD)

Alzheimer′s disease (AD) is one of the most common neurodegenerative disorders in the elderly, accounting for more than 80% of dementia cases worldwide. It causes progressive mental, behavioral, and functional impairment as well as loss of learning ability [86]. The case of lipoic acid, a chemical naturally present in the mitochondria with substantial anti-inflammatory and antioxidant capabilities capable of reducing oxidative stress, exemplifies the potential of NPS to improve the efficacy of AD therapy [87]. They are designed in such a way that they are harmless, biodegradable, and target-specific [88]. In the context of treating Alzheimer′s disease, these nano systems could effectively retain and distribute medicines and other neuroprotective chemicals to the brain [89]. The intranasal method helps to bypass the BBB, allowing medications to be delivered directly to the brain. Endocytosis, such as receptor-mediated endocytosis, phagocytosis, and pinocytosis, are the most frequent processes for nanoparticle transportation, with receptor-mediated endocytosis being the most preferred approach [90].

Curcumin is formed from the rhizome of *Curcuma longa*, cultivated in South Asian countries, especially India and China. Curcuminoids are widely used in herbal medicine for the treatment of various disorders. Recent studies revealed that curcumin plays an essential role in the management of AD, mainly acts as an essential diagnostic agent, multi target-directed drug, as well as promotes lifelong nutraceuticals [91]. Several nano-formulations have been recognized as theranostic agents that can improve the pharmacokinetic properties of curcumin and other bioactive substances, which can help overcome diagnostic and therapeutic limitations. Nanocarriers have proved to be effective in delivering curcumin and other nutritional components across the BBB, allowing them to be distributed more efficiently throughout the brain [92]. Diffusion and erosion or degradation processes convey the integrated medication to the targeted site. Liposomes, polymeric nanoparticles, micro-and nano-emulsions and dendrimers are some of the most commonly utilized nanoparticles [90].

**Figure 2:**
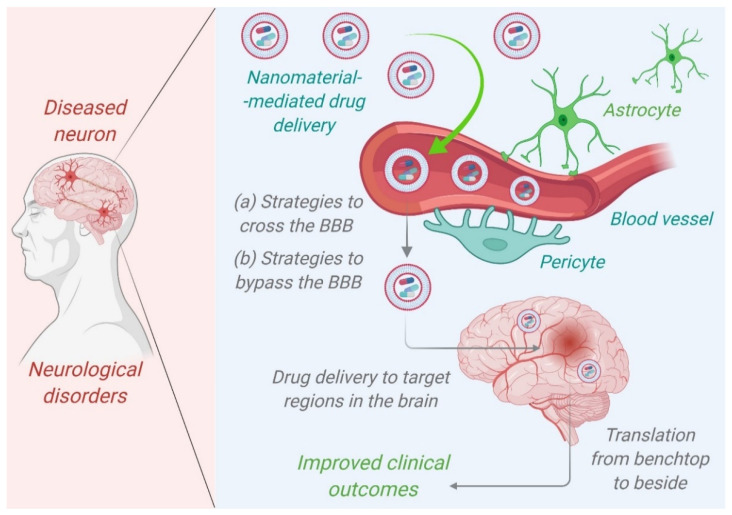
Nanomaterials mediated drug delivery of therapeutic agents targeting the brains of patients suffering from Alzheimer’s disease to improve clinical outcomes. Adopted from [99].

The type of the portion forming the outermost layer determines whether polymeric nanoparticles are hydrophilic or hydrophobic. Their delivery to the target region can be accomplished through receptor-mediated endocytosis or endothelial cell transcytosis. Drug absorption can be improved by coating PNPs with antibodies or PEG (polyethylene glycol), especially when the drug is delivered intranasally. When coated with polysorbate-80, poly(n-butyl cyanoacrylate) loaded with the drug Rivastigmine exhibited enhanced drug delivery in the case of AD [93]. Aβ1-42 monoclonal antibody-decorated nanoparticle-based treatment against AD completely corrects the memory deficit in an experimental AD animal [92]. Liposomes are biodegradable delivery nanosystems that can encapsulate a wide range of hydrophilic and hydrophobic biopharmaceuticals, including proteins, peptides, RNAs, and small molecules, without changing their characteristics [93]. The quick penetration of therapeutic biomolecules into the brain is aided by their characteristic phospholipid bilayer architectures, which resemble biological membranes. Liposomes are one of the most studied and clinically recognized nanostructured carriers due to their low toxicity, extensive track record, and ability to carry both hydrophilic and lipophilic compounds [94]. Figure 2 represents nanomaterials mediated drug delivery of therapeutic agents targeting the brains of patients suffering from Alzheimer’s disease. Dendrimers are also one of the promising NPS in the near future for the treatment of AD because of their high drug loading capacity, which includes both the inner chamber and the dendrimer′s outer surface. The size of dendrimers can be easily controlled by carefully selecting monomers and the degree of polymerization [93]. In comparison to the normal aging brain, the formation of senile plaques in AD is characterized by a higher concentration of soluble and insoluble A-42, more alterations of terminal amino acids of A and Aβ-40-42, and a rise in their concentration. The delayed accumulation of Aβ promotes pathogenic peptide changes such as racemization, isomerization, cyclization, and oligomerization, resulting in Aβ-42 insolubility [95].

PAMAM dendrimers were first reported in the 1980s and are also the first family of dendrimers to be fabricated and commercialized as well as the most studied. PAMAM dendrimers have a diameter of about 1–14 nm. These dendrimers have many applications in the field of biomedical sciences, including cargo and drug delivery, gene transfection, imaging agents, and act as potential antimicrobial agents [96]. Dendrimers PAMAM3, 4, and 5 have been demonstrated in studies to suppress the production of amyloid deposits. In comparison to other kinds of aggregated proteins, they have hydrolytic characteristics. There were no forms of Aβ resistance to hydrolysis after incubating cells with dendrimer nano-molecules. This finding suggests that PAMAM dendrimers are not only amyloid aggregation inhibitors but also have the potential to eliminate hazardous types of aggregated proteins [97]. The PAMAM dendrimers can reduce the effective concentration of amyloid formations in three ways: (a) dendrimers bind peptides, (b) increasing dendrimers locks the free ends of the fibrils, and (c) dendrimers accelerate fibril disintegration [98].

### 3.2. Parkinson Disease

Parkinson’s disease (PD) is a customary neuro-degenerative disorder besides Alzheimer′s disease [100]. PD emerges from the loss of neurons that make dopamine in the basal ganglia and substantia nigra of PD patients [101]. Signs and symptoms of PD include muscle rigidity, tremors, rigidity, sluggish motion, and postural instability. Other prevalent motor symptoms include the freezing of gait, modified gait patterns, and a lack of motor coordination [102]. Conventional medications have a lot of adverse effects and are not as bioavailable in the brain. As a result, essential measurements are required to address the therapeutic constraints of Parkinson′s disease. Nanotechnology has made a significant addition to our understanding of Parkinson′s disease pathophysiology. Nanotechnology has the potential to produce effective medicines with fewer side effects and greater bioavailability in the brain [103].

Previous research has shown that the BBB expresses a variety of receptors, including low-density lipoprotein (LDL) and low-density lipoprotein related protein-1 and 2 (LRP-1,2) transferrin and the P-gp efflux transporter. Endocytosis at the luminal side of the BBB is mediated by ligand-receptor contact, which is followed by translocation across the endothelial cytoplasm to the abluminal side, and ultimately exocytosis, which allows payload release at the target region [104]. A drug or a gene could be one of the payloads. Gene therapy is becoming a popular technique for treating Parkinson′s disease; however, there are several limitations in the currently available therapies, such as the gene vector′s limited permeability through the BBB after intravenous delivery restricts therapeutic efficacy. Due to the high invasiveness and repetitive injection schedule, local intracerebral administration of the gene vector at the target region is frequently limited by patient compliance. Schlachetzki and colleagues discussed trans-vascular delivery, a unique method for gene therapy to deliver targeted nano-carriers to the brain [105]. Because nanoparticles (NPs) can shield encapsulated drugs against biological and/or chemical degradation, as well as extracellular transport via P-glycoprotein efflux, they may increase nose to brain drug delivery. This would boost medication availability in the central nervous system (CNS) [106]. Kaili Hu et al. constructed the lactoferrin (Lf) conjugated polyethylene glycolpolylactide-polyglycolide (PEG-PLGA) nanoparticle (Lf-NP), which was used to evaluate the in vitro and in vivo delivery properties of a novel biodegradable brain drug delivery system. The behavior, immunohistochemistry, and transmitter content revealed an IV injection of 28 g UCN transported by Lf-NP might successfully attenuate the 6-OHDA-induced striatum damage. In the rat 6-OHDA Parkinson′s disease models, intracerebral injection of UCN was previously shown to stop the development of Parkinsonian-like symptoms. Noninvasive systemic administration was still impossible due to UCN′s limited BBB permeability. This study was the first to show that noninvasive delivery of UCN to the brain was possible and that the Lf-NP delivery system may be used for noninvasive Parkinson′s disease treatment [107]. Autopsy specimens collected from the brains of deceased PD patients revealed unique protein clumps called amyloid fibrils and Lewy bodies within the brain tissue [108]. These aggregates are dense protein polymers made up of monomers of α-synuclein. α-synuclein is a member of the synuclein family with 140 amino acids that plays a key role in neuronal signaling and intracellular processes [109].

**Figure 3:**
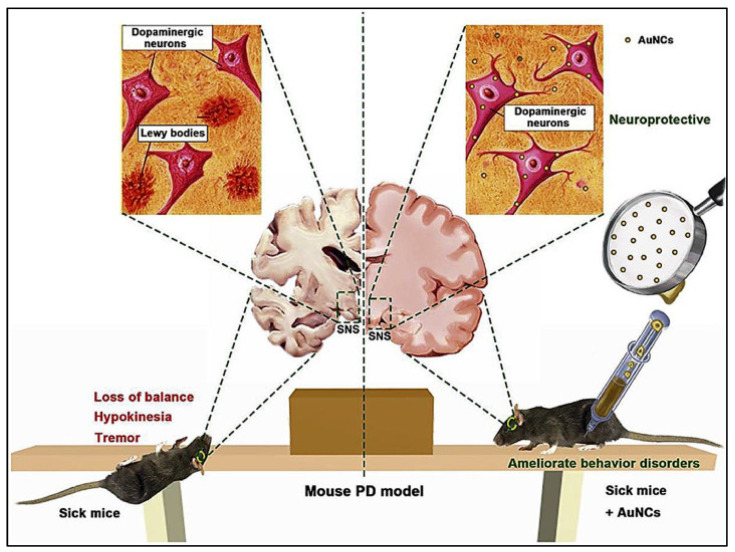
Schematic diagram of the possible mechanisms for the treatment of PD. Adopted from [111]. Copyright Elsevier 2021.

In recent years, there has been a lot of interest in looking into the potential of different nanoparticles for preventing the production of α -synuclein amyloid. Several nanoparticles have been studied for this purpose, including gold nanoparticles, super paramagnetic iron oxide nanoparticles, QDs graphene, and graphene derivatives [110]. The CNTs interacted strongly with α-synuclein, with the phosphorus-doped CNTs exhibiting the strongest connections. Doped-CNTs, particularly phosphorus-doped carbon nanotubes, have been shown to suppress the development of α-synuclein amyloid efficiently and hence could be considered as a potential Parkinson′s disease treatment. However, additional in vitro and clinical research is required. Direct contact with a misfolded α-synuclein protein can cause the misfolding of other α-synuclein proteins, exacerbating the condition. As a result, α-synuclein misfolds prevention may be a viable PD therapeutic option [110]. The schematic illustrations are shown in Figure 3.

### 3.3. Huntington Disease

Huntington′s disease is a progressive neurodegenerative disease with a specific phenotype that includes chorea and dystonia, incoordination, cognitive impairment, and behavioral issues. Symptoms usually appear in middle age after a person has had children; however, the disease can appear at any period between childhood and senescence [112]. An enlarged CAG trinucleotide repeat (of variable length) in the gene that codes for the huntingtin protein (HTT) causes it. Epigenetic modifications have been linked to the development of HD. Transcriptional dysregulation is a key pathogenic mechanism in HD that connects dietary availability to cellular activities. Mutant HTT aggregates may result in dramatically reduced histone acetylation, associated with neuronal injury and loss in HD [113].

**Figure 4:**
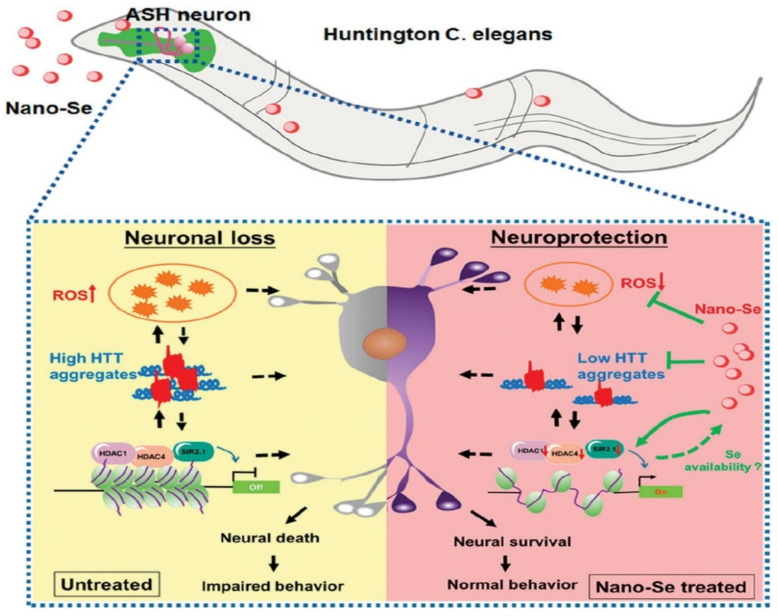
Schematic diagram of the neuroprotective effect of nano-Se in a model of *C. elegans* HD. Reproduced with permission [116]. Copyright 2019 The American Chemical Society.

Nano-based techniques have had a significant impact on neurodegenerative disease prevention. The design and engineering of nanoparticles have an impact on protein fibril nucleation, degrade mature protein fibrils, and target aggregated protein plaques via the BBB [114]. Selenium (Se) is an essential trace element for both animals and humans. It is also needed for regular brain function and has neuroprotective properties. Seleno proteins have been discovered to play a role in regulating neurodegenerative disorders, including Alzheimer′s disease [115]. Similarly, nano-selenium is a promising antioxidant, anti-aging, and has high drug loading efficacy. SeNPS has been evaluated using animal models and the authors documented that doses less than 20 mM had no significant harmful effect on *Caenorhabditis elegans* (*C. elegans*) in the HD model experiment. At a dose of 2 mM, nano-Se efficiently reduced the oxidative stress generated by juglone and methylmercury (MeHg) in *C. elegans* without compromising their growth. Two mM Selenium NPS therapy boosted the survival rate of ASH neurons in transgenic HA759 worms to 44%, and significantly improved the recovery of physiological activities in ASH neurons, such as a proper response to external stimuli. Antioxidative nano-Se was thought to serve a key role in controlling the production of histone deacetylase (HDAC) members and efficiently reducing HTT aggregation [116]. A schematic diagram of the neuroprotective effect of nano-Se in a model of *C. elegans* HD is shown in Figure 4. The poly(trehalose) nanoparticles are formed of a 6 nm iron oxide core and a zwitterionic polymer shell containing 5–12 wt percent covalently bonded trehalose and have a hydrodynamic dimension of 20–30 nm. The nanoparticle form of trehalose with a zwitterionic surface charge and a trehalose multivalency (i.e., number of trehalose moles) is 1000–10,000 times more efficient than molecular trehalose in inhibiting protein fibrillation in extracellular space, blocking the aggregation of the polyglutamine-containing mutant huntingtin protein in model neuronal cells, and suppressing mutant huntingtin aggregates in HD mouse brain [114].

Biocompatible and non-toxic poly(lactic-co-glycolic acid) nanoparticles (PLGA NPs) offer several advantages, including greater drug solubility, resistance to enzyme digestion, increased targeting efficiency, and improved cellular internalization [117]. The most abundant polyphenol in the tea plant, epigallocatechin-3-gallate (EGCG), has previously been found to have positive benefits in Huntington′s disease (HD), but its therapeutic stability restricts its therapeutic efficacy. EGCG encapsulation in poly(lactic-co-glycolic) acid-PEG nanoparticles (NPs) increased EGCG stability and brain penetration, as well as the therapeutic effects in other ND. To test the possible usefulness of the proposed nano-carrier in this ND, EGCG-loaded NPs were given to 3-nitropropionic acid (3-NP)-intoxicated mice, which is a well-known animal model of HD [118]. Thus, research in this field is quite fascinating in order to increase the therapeutic armament, particularly through the use of PLGA NPs [117].

### 3.4. Amyotrophic Lateral Sclerosis (ALS)

The progressive loss of motor neurons in the brain and spinal cord is a hallmark of Amyotrophic lateral sclerosis. The pathobiological aspects of this neurodegenerative illness are similar to those of front temporal dementia, and many individuals exhibit symptoms of both diseases. Various genes and pathophysiological processes cause the disease, and it will be required to comprehend this heterogeneity to develop effective treatments [119]. The development of diagnostic techniques for the early detection and successful treatment of various illnesses has been the subject of extensive research. However, just a small amount of progress has been made. The BBB, which prevents therapeutic drugs and diagnostic equipment from penetrating the brain, remains one of the reasons for the lack of success in the development of treatments to date. There are few techniques for treating neurodegenerative illnesses that are nearing completion, including the use of stem cells and antitoxins against mutant forms of the copper and zinc superoxide dismutase (SOD1), as well as nanotechnology [120].

Since oxidative stress plays a role in amyotrophic lateral sclerosis (ALS) in humans, the SOD1 Cerium oxide nanoparticles (CeNPs) are a type of cerium oxide that can neutralize reactive oxygen and nitrogen species. Because oxidative stress is linked to amyotrophic lateral sclerosis (ALS) in humans and the SOD1 G93A mouse model of ALS, CeNPs was tested in SOD1 G93A transgenic mice, which would increase their survival and reduce disease severity. SOD1 G93A mice were given CeNPs twice a week starting at the onset of muscular weakness, which preserved muscle function and enhanced longevity in both males and females [121]. According to research, only three attempts have been made to produce an ALS therapy that combines nanotechnology with riluzole medication. Bondì et al. created solid lipid nanoparticles containing riluzole for ALS treatment. Because of their lipophilic characteristics, these nanoparticles could target the brain via endocytosis. The rats were given riluzole-loaded nanoparticles and regular riluzole separately, and the results were compared. Compared to traditional riluzole, the riluzole-loaded nanoparticles were able to bypass the BBB and transport more medicine to the brain [120].

In another study, Solid Lipid nanoparticles (SLNs) were used as carriers for riluzole. Compritol^®^ 888 ATO was used as the lipid matrix, phosphatidylcholine as the surfactant, taurocholate sodium salt as the cosurfactant, and riluzole-loaded SLNs were successfully synthesized utilizing the microemulsion process. Under various physiological settings, the systems were characterized in terms of particle size, z-potential, and drug-release profile. The blood and tissue temporal bio-distribution of riluzole, free or blended into SLNs at equivalent dosages (8 mg/kg), was investigated after intra-peritoneal treatment to rats in order to demonstrate the preferential accumulation of riluzole-loaded SLNs into the brain over water dispersion of riluzole. The drug was successfully transported into the CNS using solid lipid nanoparticles. When riluzole was given as drug-loaded solid lipid nanoparticles, the drug bio-distribution in organs such as the liver, spleen, heart, kidneys, and lung was low [121].

**Figure 5:**
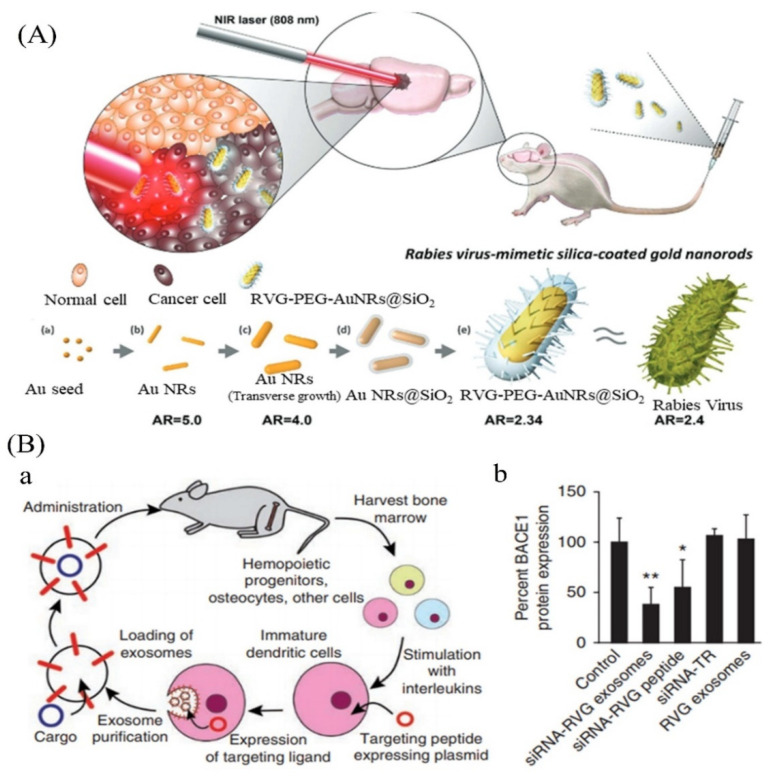
(**A**) Rabies virus mimicking silica-coated gold nanorods bypass the BBB via neuronal pathways to treat brain disease. (**B**) Delivery of therapeutic siRNA to the mouse brain by systemic injection of exosomes. (**a**) Schematic illustration of the preparation of exosomes; (**b**) gene silencing efficiency by different vehicles. Adopted from Wang et al., 2020 [123].

Exosomes are natural nanomaterials based on internal membranes that have demonstrated significant advantages over other nanomaterials due to their non-immunogenic properties and capacity to carry a range of payloads [122]. Exosomes, for example, have been produced by Lydia et al. to carry siRNA into the brains of mice. After activation, exosomes were isolated from dendritic cells collected from bone marrow with interleukins to minimize immunogenicity. To improve brain targeting, dendritic cells were modified to express Lamp2b, an exosomal membrane protein linked to the neuron-specific RVG peptide [123]. In the mouse brain, the manufactured exosomes could carry siRNA to microglia, neurons, or oligodendrocytes, resulting in cell-specific gene suppression [124]. Exosomes were created by human mesenchymal stem cells (MSCs) and treated with interferon-gamma (IFN-) ((IFN-) was utilized to increase the production of numerous critical immunosuppressive cytokines in MSCs in another investigation). These exosomes were administered intravenously in an autoimmune encephalomyelitis (EAE) animal model after being loaded with anti-inflammatory, neuroprotective RNA, and protein components, demonstrating strong BBB penetration and encouragingly regaining motor abilities [123]. Because exosome delivery systems have a high level of biological tolerance, they should be able to move quickly into clinical trials. As a result, novel exosome nanotechnology has the potential to transport several therapies over the BBB to treat a variety of neurological illnesses, including ALS [125]. The detail is shown in Figure 5. Glucose transporter-1 (GLUT1) is substantially more highly expressed in BCECs than several other transporters and receptors. Researchers recently used this knowledge to improve nanoparticle delivery over the BBB in an animal model [123]. The goal of Wiley et al. was to show that injecting nano-encapsulated minocycline intracerebroventricularly increased the site-specific activity of the medication, thereby delaying the onset of ALS and lengthening the lifetime of SOD1 animals [126].

### 3.5. Frontotemporal Dementia

Frontotemporal dementia is an umbrella clinical term for a collection of neurodegenerative illnesses marked by progressive abnormalities in behavior, executive function, and language. Frontotemporal dementia is a prevalent type of dementia that affects people under 65 [127]. A nanotechnology-based drug delivery platform offers prospective therapeutic methods for treating several common neurological illnesses, including frontotemporal dementia, among innovative strategies to overcome these restrictions and successfully deliver medications to the CNS [128].

Curcumin′s limited absorption, high metabolism, and rapid excretion make it challenging to employ in vivo. To overcome these limitations, various techniques might be used. In a neural cell line containing TDP-43 mutations Q331K or M337V, the protective effect of analogue dimethoxy curcumin was demonstrated. Dimethoxy curcumin improved the transmembrane potential, increased electron transfer chain complex I activity, and up regulated UCP2 to repair mitochondrial damage (uncoupling protein 2). Cells expressing mutant TDP-43 had abnormally high excitability, which was improved by the same drug. Furthermore, monocarbonyl dimethoxycurcumin C, an enhanced curcumin analogue, inhibited mutant TDP-43 aggregation and reduced oxidative stress, presumably due to the increased production of heme oxygenase-1 [129]. Another method for increasing curcumin bioavailability is to use nanoparticles. Curcumin-loaded inulin-d-alfa-tocopherol succinate micelles were successfully transported into mesenchymal stromal cells, demonstrating the possibility for ALS treatment [130].

### 3.6. Prion Disease

Prion disorders are a set of human and animal neurodegenerative illnesses in which the prion protein plays a crucial role in their pathogenesis. Creutzfeldt–Jakob disease (CJD), Gerstmann–Straussler–Scheinker syndrome (GSS), fatal familial insomnia (FFI), and kuru are the traditional clinical classifications for human prion illnesses. They can also be classed as acquired (transmitted between animals or people), hereditary, or sporadic based on their aetiology (unknown cause). In biology, the supremacy of a single protein in a disease with such varied pathways is unprecedented [131]. There is currently no effective treatment for prion diseases in humans, and these diseases are usually deadly in people. Drugs that demonstrate some efficacy in treating prion disorders in tissue culture systems or whole animal systems have been found [132]. There is a legitimate need to develop medication or alternative therapy for prion diseases, given the existing state of the treatment plan. As a result, Michal Mizrahi et al. investigated whether pomegranate seed oil (PSO) in NE form slows clinical progression, neurodegenerative pathological characteristics, and prions in TgMHu2ME199′K mice. E200K PrPmutation was connected to this mouse model. In mouse models with the idea of fight ND, it was discovered that PSO administration delayed the commencement of action [133]. Nano-PSO was effective in the prevention and therapy of a genetic prion disease model, suggesting that chemicals that inhibit lipid oxidation could be helpful for a variety of neurodegenerative disorders [134].

Prefibrillar amyloid formations, such as spheroidal aggregates (nanoparticles and nanospheres), have been hypothesized to be highly cytotoxic in many neurodegenerative disorders, similar to the molecular oligomers discussed above [135]. Large prion protein (PrP) aggregates were degraded into smaller PrP nanoparticles with diameters ranging from 17 to 27 nm by Silveira and colleagues, and then PrP nanoparticles were subsequently studied with DLS, non-denaturing gel electrophoresis, and transmission electron microscopy (TEM) [136,137]. Their findings revealed that the most infectious initiators for prion disorders are PrP nanoparticles, with masses corresponding to 14–28 PrP molecules. In another investigation, Moustaine et al. created amyloid nanofibrils and nanoparticles from recombinant PrP under high pressure [136].

### 3.7. Spinal Cord Injury

Spinal Cord Injury (SCI) is a neurological disorder caused by a traumatic injury or disease that affects voluntary motor control and sensory function and the autonomic nervous system, impacting cardiovascular, cognitive, bladder, and bowel functions, among other things. Changes in sexuality, weight gain, poor sleep, diminished cognitive performance, and chronic pain is common psychosocial issues for SCIs: relationship stress and breakup, societal prejudice, and reduced work possibilities [138]. Although spinal cord injury (SCI) is not as prevalent as other injuries, it has devastating physical and emotional implications. After a spinal cord injury, only a small percentage of people recover completely [139]. Applied neuroprotective techniques have been attempted in SCI models in the hopes of preserving neuronal and glial cell populations by targeting one or more of the aforementioned secondary damage events through drug-mediated protective effects. However, few pharmaceutical treatments have been successful in translating therapeutic value in patients [140].

At pH 7.4, micelles encapsulating 1,2-benzisoxazole-3-methanesulfonamide with a size of about 60–90 nm enabled a prolonged drug release. It was discovered that drug-loaded micelles had a more significant protective impact against neurotoxicity than non-formulated species [141]. Various neuroprotective regimens, such as maintaining cyclic adenosine monophosphate (cyclic AMP) levels with injection of the phosphodiesterase 4 (PDE4) inhibitor Rolipram (Rm), can lessen the extent of damage following SCI [142]. Rm, like many other medications, is relatively hydrophobic and has a low aqueous solubility (0.2 mg/mL) [143]. Mack et al. (2018) created the PgP[poly(lactide-co-glycolide)-graft-polyethylenimine] polymeric micelle nanoparticle as a carrier for rolipram in SCI improvement. PgP has been constructed and polymerized to deliver therapeutic nucleic acids and medicines for SCI repair in a combinational delivery system. PgP has a hydrophobic core and a hydrophilic shell, allowing rolipram and small-interfering RNA to be carried to the SCI site [144].

Silica nanoparticles (SiNPs), which have been shown to be non-toxic in vivo, have also been explored extensively for the treatment of SCI. In ex vivo and in vivo contusion guinea pig models of SCI, Cho et al. established the efficacy of PEG decorated SiNPs (PSiNPs). The NPs, in this case, are not carrying any drugs; instead, they are increasing the bioavailability of PEG, which has been shown to have neuroprotective properties and closes broken cell membranes [145]. When NPs were used instead of PEG alone, the effective concentration of PEG was reduced by two orders of magnitude. This is crucial because PEG has been demonstrated to be beneficial for treatment. Its administration is restricted by viscosity and the concentration of PEG monomers, which can be poisonous at large doses [146,147]. Because of their favorable qualities, such as free radical scavenging or the capacity to traverse the blood spinal cord barrier (BSCB), several other lesser-known NPs are being investigated in the early stages of treating SCI. Poly(butyl cyanoacrylate) NPs (PBCANPs) coated with the surfactant polysorbate-80 have been demonstrated to cross the BBB in previous experiments [148,149]. These particles are covered with adsorbed plasma proteins, including apoplipoprotein E, and it is thought that they are mistaken for low-density lipoprotein particles and ingested by the low-density lipoprotein uptake system, allowing them to pass across the BBB [149].

**Figure 6:**
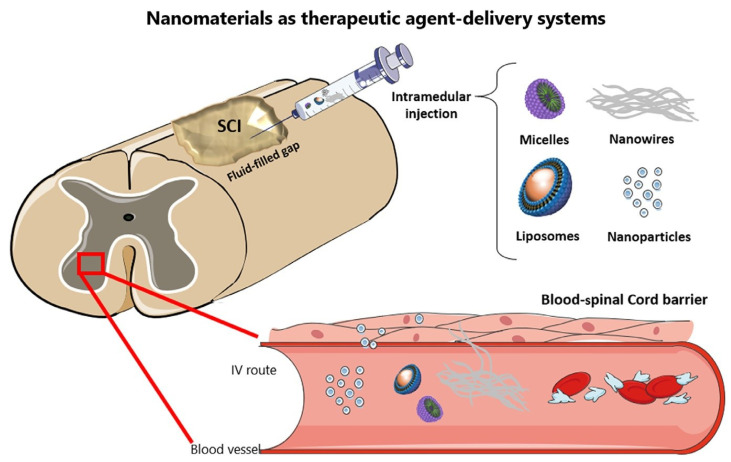
Different nanomaterials as drug delivery systems for the treatment of SCI. One of their most important features is their small size (nanoscale), which makes them capable of crossing the blood spinal cord barrier. Adopted from [151], with permission from Elsevier Limited 2019.

Aside from enhancing BSCB crossing, nanoparticles, micelles, and liposomes might boost the bioavailability of various therapeutic medications over long periods, avoiding the numerous adverse effects now associated with drugs used in clinics. On the other hand, nano-electrospun fibers, SAP fibers, and nanotubes have a lot of potential since they can replicate cells′ physical and structural structure and the ECM. This improves the material′s affinity for neurons and axons, resulting in appropriate substrates for neural regeneration. Several issues still need to be addressed, including the clearance of these nanosystems as well as the still small gains seen in preclinical animals. Figure 6 represents different nanomaterials as a drug delivery system for the treatment of SCI. Future research should concentrate on merging nanotechnologies with other treatments, as this is the only approach to address a condition as complicated as SCI [150].

### 3.8. Stroke

Stroke is a prominent cause of mortality and disability globally. It can be divided into two types: ischemic stroke and haemorrhagic stroke, including intracerebral and subarachnoid haemorrhage [152]. Pioneering studies in the 1970s discovered that the ischemic penumbra, a hypoperfused, hibernating, electrically non-functional portion of the brain, is responsible for much of the early clinical deficiency in stroke patients [153]. Stroke is a major public health issue that affects people all over the world. Approximately one-third of stroke victims die within a year, and a similar number of patients are permanently incapacitated [154]. Over decades, scientists have failed to convert over 1000 experimental medicines discovered in cells and animals into human use [155,156]. The use of nanotechnology in cell therapy and tissue engineering has the potential to improve the treatment of brain and spinal cord injuries in the future. Stem cells have been found to increase the functional recovery by selectively targeting the injured brain and spinal cord tissue [157].

PLGA, a biodegradable polymer with hydrolyzable ester linkages, is a common nanocarrier. The polymer is hydrolyzed, resulting in a reduction in molecular size and the formation of gaps between polymer chains, which aids the drug release. It is the best choice for biomedical applications because both the hydrolysis product and the polymer itself are harmless [158]. Liposomes, self-assembling vesicular carriers made from amphipathic lipids, are among the most commonly used lipid-based carriers [159]. Liposome surface modification and lipid composition changes can assist change in the drug release profile. This can also boost its distribution and bioavailability in other tissues, as well as its lifespan at the targeted location [158,160]. Surface modification of the lipid surface with PEG (e.g., PEG monostearate) delayed opsonization and increased liposome lifetime [161,162]. Stimulus-responsive liposomes have also been produced, releasing therapeutic drugs for stroke in a controlled manner [161]. Various liposomal systems have been described for the treatment of ischemic stroke. Phosphatidylserine (PS), dioleoylphosphatidylethanolamine (DOPE), cholesterol, and di-stearoyl phosphoethanolamine-polyethyleneglycol-2000 are the primary components of liposomes (DSPE-PEG-2000) [163]. Various functional nanoparticles have been designed as promising drug delivery platforms that are expected to improve the therapeutic effect of ischemic stroke, inspired by the prominent merit of nanoscale particles in brain targeting and BBB penetration [164]. Perfluorocorban Nanoparticles (PFC-NPS) are non-metabolizable, non-toxic, and inert. PFC-NPS have the ability to target epitopes while also being integrated onto the NP surface and carrying a high paramagnetic payload of 60,000 to 90,000 gadolinium ion per particle. Therefore, it is a very sensitive detector to target epitopes with MRI. Early manifestations were detected for αvβ3-integrin-targeted PEC-NPS along with MRI. The magnitude of the MRI signal is proportional to the neurovascular density, and PFC-NPS can increase this magnitude. Ligand-mediated PFC-NPS show promise for detecting therapeutic responses in stroke patients early and accurately [165]. Deep Vein Thrombosis (DVT) and Pulmonary Embolism (PE) are serious stroke complications [166].The surgical procedures of thrombin injection in the middle cerebral artery or clot injection in the carotid artery involved in embolic stroke models in thrombolytic research are too sophisticated. Haoyuan et al. present a new mouse stroke model that embolizes magnetic nanoparticles (MNP) cross-linked with thrombin. MNP@Thrombin was injected from the tail vein after the magnet was placed in the common carotid artery. The MNP@Thrombin accumulated in the carotid artery and caused thrombus formation within minutes of injection. These complex clots were flushed into the cerebral artery and subsequently blocked it [167].

**Figure 7:**
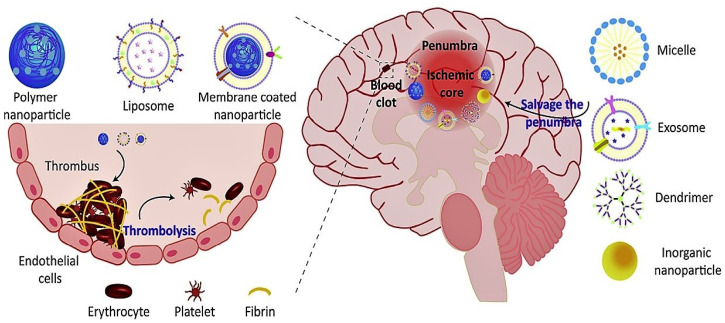
Schematic diagram of different nanomaterials for the treatment of stroke. Adopted from Chao Li et al., 2020 [163].

More than 200 nano-medicines based products have been approved for clinical use or are accurately studied in clinical trials. Liposomes are the most widely studied nanomedicine method and have been approved by FDA (e.g., Doxil, Ambisome, DepotDur, and DaunoXome) [166]. A few examples of nano-based drugs approved by the FDA for the treatment of neurological disorders are shown in Table 1. Different nanomaterials used for the treatment of stroke are shown in Figure 7.

### 3.9. Brain Tumors

A brain tumor can affect both adults and children and is caused by various cell types that give rise to tumors with varying degrees of malignancy and invasiveness [175,176]. Brain tumors are a diverse group of neoplasms that are typically classified as benign or malignant. The leading causes of brain tumor malignancy are thought to be uncontrolled proliferation and tissue infiltration of dedifferentiated cells caused by harmful chemical, physical, and biological exposures [177]. CNS tumors are the most common solid tumor and the leading cause of death from cancer in children, adolescents, and young adults, with an incidence of 1 to 5 cases per 100,000 people [178]. Surgery, radiation, and systemic chemotherapy are common treatments for brain tumors, but they are associated with a high recurrence rate and a poor prognosis. Intra-arterial administration of anti-cancer drugs has been considered a viable alternative to intravenous and oral administration in recent decades [177]. Liposomes, nanoparticles, drug-loaded micro bubbles, and magnetic attraction of cells are examples of these strategies [178].

**Figure 8:**
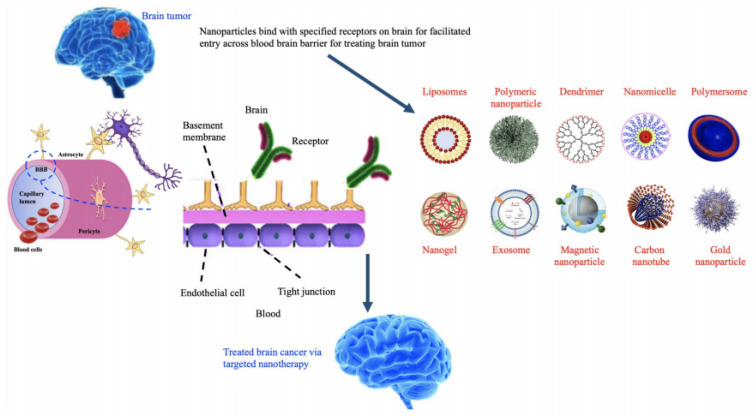
Nanomaterials for the treatment and diagnosis of brain tumors. Adopted from Simona et al., 2020 [183].

NPs, on the other hand, have been shown to have a therapeutic advantage in delivering potential antitumor drugs in several studies [179]. PH-responsive nano-drug delivery systems, nanosomes, and exosomes loaded with therapeutic drugs or RNAi make up the nanomedicine preparations [180]. The use of polymeric NPs to deliver small interfering RNA to several genes, including sodium-potassium (Na–K)–chloride cotransporter 1, yes–associated protein 1, roundabout homolog 1, EGFR, and survivin, can significantly reduce glioblastoma cell growth and migration in a selective manner [181,182]. Different types of nanomaterials with their mechanism of action are shown in Table 2 and Figure 8.

## 4. Conclusions

According to the Global Burden of Disease studies and the World Health Organization, neurological disorders cause approximately 276 million disabilities with 9 million mortalities throughout the globe each year [9,193]. At the same time, these cases are expected to increase tremendously. NDs are difficult to treat and manage due to the BBB, a physiological interface that limits the entry of various therapeutic agents into the brain, which is one of the most critical issues in the treatment of NDs. Therefore, there is a need for brain-targeted drug carriers to treat NDs. Due to the inability to cross the BBB and the lower efficacy of traditional drugs, the nano-scaled drugs delivery system has unique and versatile properties, which attracts scientists from different areas of biomedical science, especially neurosciences, due to its unique characteristics, including nano range size, high surface to volume ratio, selectivity, sensitivity, surface modification, charges, and stability. The nano-based approach represents an innovative and promising strategy for the treatment and diagnosis of various diseases such as cancer and NDs. Different types of nanomaterials are widely used as potential candidates for the imaging and treatment of NDs. In recent years, the development of nanoparticles mediated delivery of drugs have showed a potential approach to cross the BBB, reach the CNS, and perform their function.

The potential use of various types of nanomaterials and nanoparticles for the treatment of various types of NDs, especially PD and AD, has been widely investigated. However, these nanotherapeutic approaches also have several issues. The main issue is the safety concern, because the degradation pathways of various nanomaterials and their toxicity in the brain have not been fully understood. Therefore, further studies are needed to fully elucidate these issues. The second issue is the complex pathogenesis of neurological diseases. These diseases′ clinical and pathological features may vary in different disease stages. Therefore, the animal models currently used for these diseases do not fully mimic the pathology of the disease. Hence, the animal model selection is noteworthy. The major and most challenging issue in this area is clinical translation. Many studies are in the in-vitro stage or at the stage of the animal model. Only a limited number of drugs are approved by the FDA and/or are under various stages of clinical trials. There is a lack of clinical trials for nano-based approaches for NDs treatment because the potential side effects of these strategies in humans are still largely unknown. Similarly, The pharmacodynamic and pharmacokinetics of these nanomedicines are still unknown and limited. Therefore, the translation of these results into a clinical application is still a big challenge in the area of nanotherapeutic strategies, and it will be the main focus of nanomedicine research in the next decade. As the nanomedicine field is still new and not fully explored, from bench to clinical trials, rigorous protocols of validation of in vitro as well as in vivo protocols are required. Innovation from engineers, chemists, and other researchers in the field is required for large-scale production. Similarly, to facilitate access to the trails and patients, the adaptation of regulatory policies is necessary. More research is required to understand the nanoparticles′ safety concerns and validate their therapeutic applications.

## Figures and Tables

**Table 1:** Examples of FDA-approved drugs for the treatment of various NDs.

Nanomaterials/Nanoparticles Used	Drug Name	Diseases	Year of Approval	Route	References
PEG-PLGA	Riluzole	Amyotrophic lateral sclerosis (ALS)	1995	Orally	[123]
Poly(n-butylcyanoacrylate)	Rivastigmine	Alzheimer’s disease	2000	Intravenous	[168]
CeO_2_ NP	L-DOPA	Parkinson disease	2003	Intranasal	[169]
(MPB-PE) and (PDP-PE) couples	D-Penicillamine	Alzheimer’s disease	2005	Nasal delivery	[170]
Gold Nanocrystals	Molecular surgery	Alzheimer’s disease	2006		[171]
Inorganic and metallic nanoparticles	Nanotherm^®^(MagForce)	Glioblastoma multiform	2010	Intravenous injection	[128]
Intracerebral Biodegradable gel matrices/Polymer nanoparticles	Temozolomide	-	2010	Orally	[172]
Liposomes/Microcapsules	lomustine	Brain Tumor	2010	Orally	[172]
CED/Intracerebral/Intraarterial/Liposomes	Carboplatin	Brain Tumor	2010	Intravenous	[172]
Polymer NP	Copaxone^®^/Glatopa (Teva)	Multiple Sclerosis	2018	Subcutaneousinjection	[128]
Liposomes and polymers	Riluzole	Amyotrophic lateral sclerosis (ALS)	2019	Intravenous	[123]
Gold nanocrystals	CNM-Au8	Multiple sclerosis	2021	Orally	[173]
PLGA	L-DOPA	Parkinson disease	-	Intranasal	[174]
Cerium oxide	Photothermal therapy	Stroke	2021	-	[26]
Thermotherapy and magnetic iron-oxide NPs + reduced dose radiotherapy	Nano-thermotherapy	Glioblastoma multiforme	Phase II clinical trials	Intrathecal	[128]

**Table 2:** Different types of nanomaterials with their mechanism of action.

NPS	Mechanism of Action	Disease	Animal Model Used	References
Polymeric NPs	Transport vectors/penetrate the cell membrane through endocytosis	Stroke	NA	[184]
Micelles	Intravenous delivery/efficient drug delivery	PD	Mouse model	[184]
Graphene NPs	Destroy cancer cells	Brain tumors	Chicken embryonic angiogenesis assay	[185]
Gold NPs	Improved selectivity to brain	AD	Mice	[186]
Carbon NPs	Platelets aggregation/stem cell therapy	Stroke	Rat	[183,187]
Silver NPs	Efficient drug delivery	Brain tumors	-	[188]
Zinc oxide	Efficient drug delivery	Brain tumors	Mature Rats	[188]
dendrimers	Efficient drug delivery	Brain tumors	Mice	[188]
Lipid NPs	Accumulation of edelfosine	Brain tumors	Xenograft mouse model of glioma	[189]
Theranostic NPs	Improved pharmacokinetics	Brain tumors	-	[190]
Cerum oxide	therapeutic effect by acting as redox active agents	ALS	Male and female mice	[191]
Magnetic NPs	Regulate the metal homeostasis in the brain, carry a large dose of drug to achieve high local concentration and avoid toxicity, target and detect amyloid plaques in AD	AD/HD/Frontotemporal Dementia	-	[192]
